# Supplementary material for: Trophoblast organoids with physiological polarity model placental structure and function
Source: J Cell Sci. 2023 Sep 7;137(5):jcs261528. doi: 10.1242/jcs.261528 (PMC10499031; doi:10.1242/jcs.261528)
Supplement: Supplementary information [file joces-137-261528-s1.pdf]

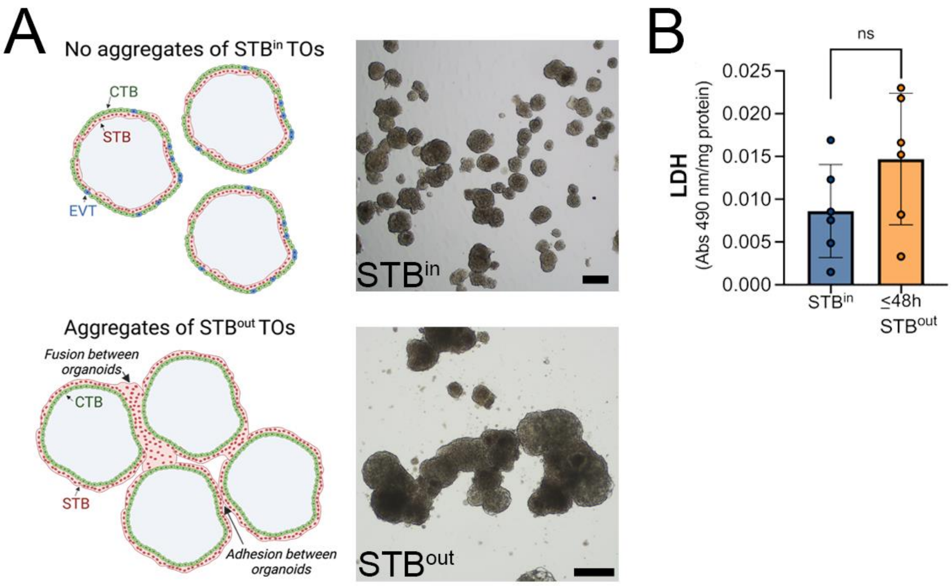

Fig. S1. Evaluation of STB^{in} and STB^{out} TOs. (A), Left, schematic of STB^{in} (top) or STB^{out} (bottom) TOs demonstrating the aggregation that can occurs in STB^{out} TOs that results from fusion of the STB and/or adhesion between organoid units. At right, brightfield images of STB^{in} (top) or STB^{out} (bottom) TOs demonstrating the extent of aggregation that can occur. Scale, 150μm (top) and 125μm (bottom). All schematics created using Biorender**. (B),** Levels of lactate dehydrogenase (LDH) in conditioned medium from STB^{out} TOs cultured for ~48hrs. Data are shown as 490nm absorbance normalized to total protein. Data are shown as mean ± standard deviation with significance determined by a student's t-test (ns, not significant). Symbols represent unique fields of organoids from individual replicates.

Journal of Cell Science • Supplementary information

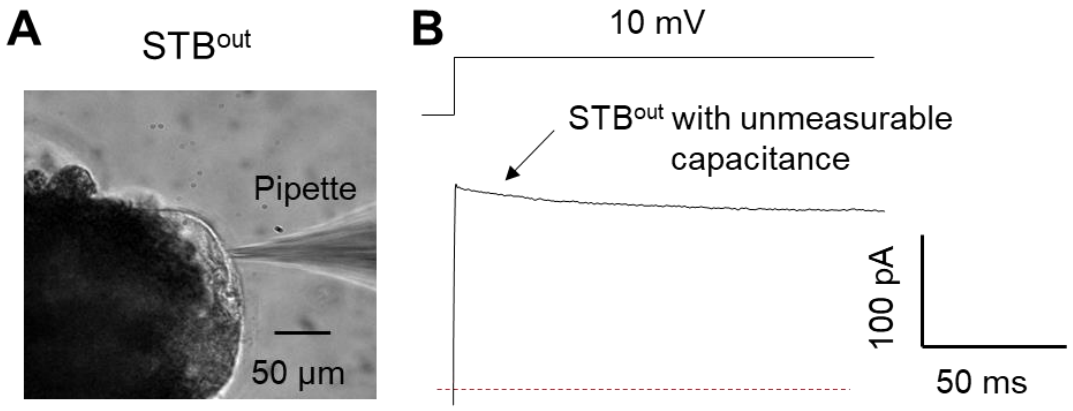

Fig. S2. Patch clamp measurement of large syncytia from the surface of STB^{out} TOs. (A), Representative brightfield image of a patch-clamped, extremely large syncytia from trophoblast organoids (TOs) growing under STB^{out} conditions. **(B),** Representative membrane test trace from a large STB. Cell capacitance cannot be accurately measured due to the space clamp issue of large syncytia. Current was elicited by a test voltage pulse of 10 mV from a holding potential of 0 mV (top).

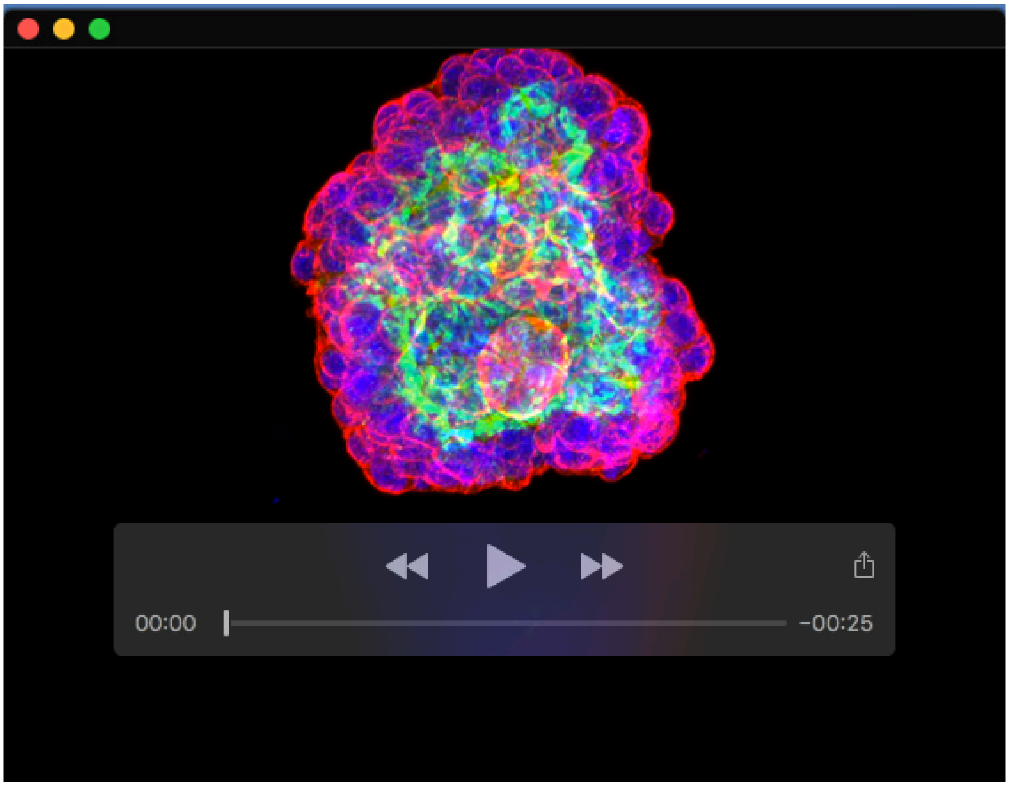

Movie 1. Three-dimensional image reconstruction of an STB^{in} trophoblast organoid (shown in Figure 2B, top) immunostained for SDC-1 (in green) and cytokeratin-19 (in red). DAPI-stained nuclei are shown in blue.

Journal of Cell Science • Supplementary information

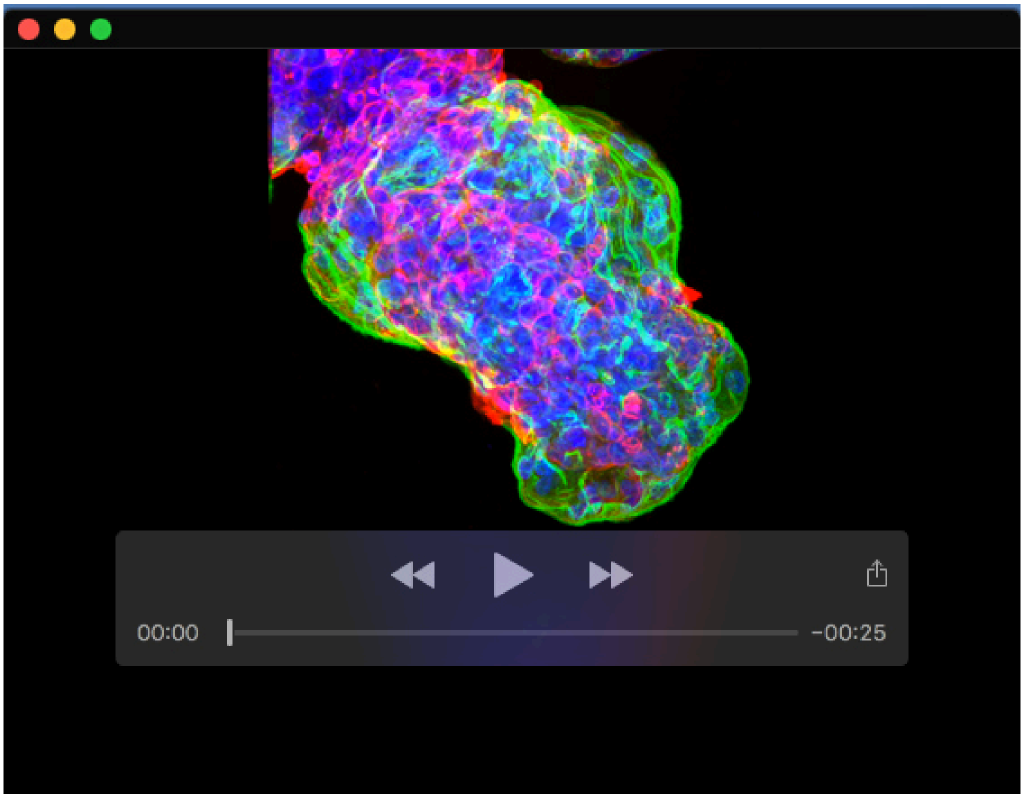

Movie 2. Three-dimensional image reconstruction of an STB^{out} trophoblast organoid (shown in Figure 2B, bottom) immunostained for SDC-1 (in green) and cytokeratin-19 (in red). DAPI-stained nuclei are shown in blue.

Journal of Cell Science • Supplementary information

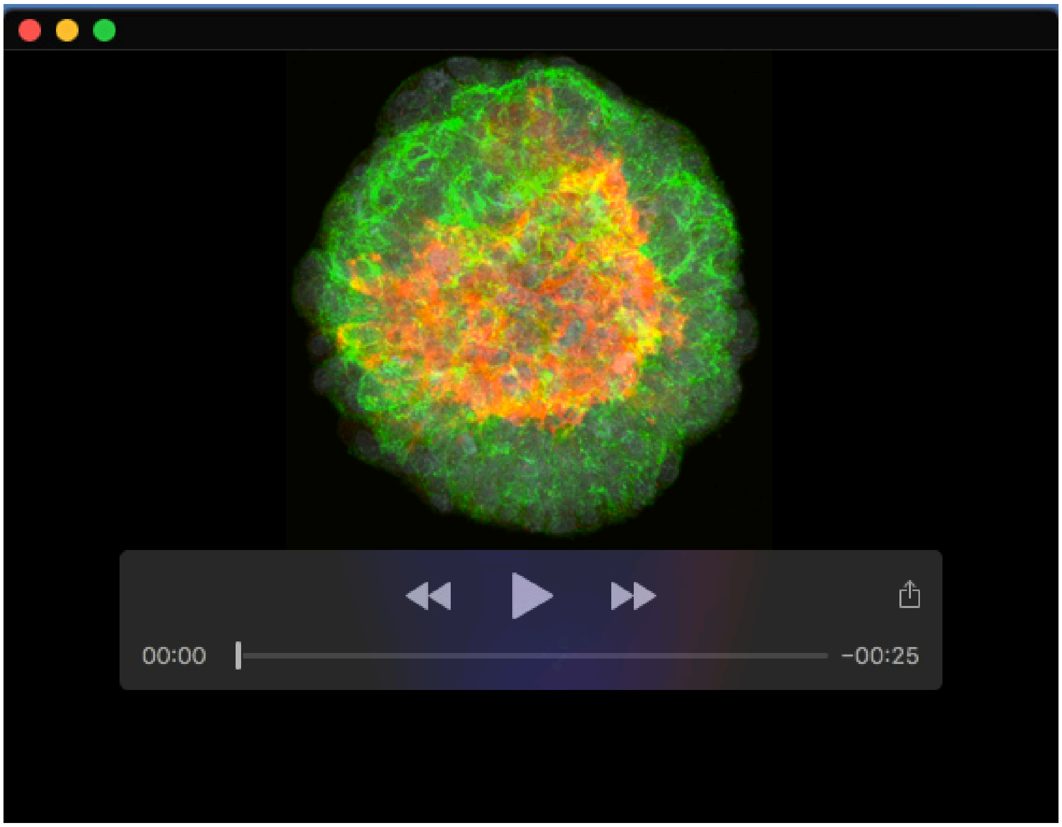

Movie 3. Three-dimensional image reconstruction of an STB^{in} trophoblast organoid (shown in Figure 2B, top) immunostained for ITGA6 (in green) and CGBs (in red). DAPI-stained nuclei are shown in grey.

Journal of Cell Science • Supplementary information

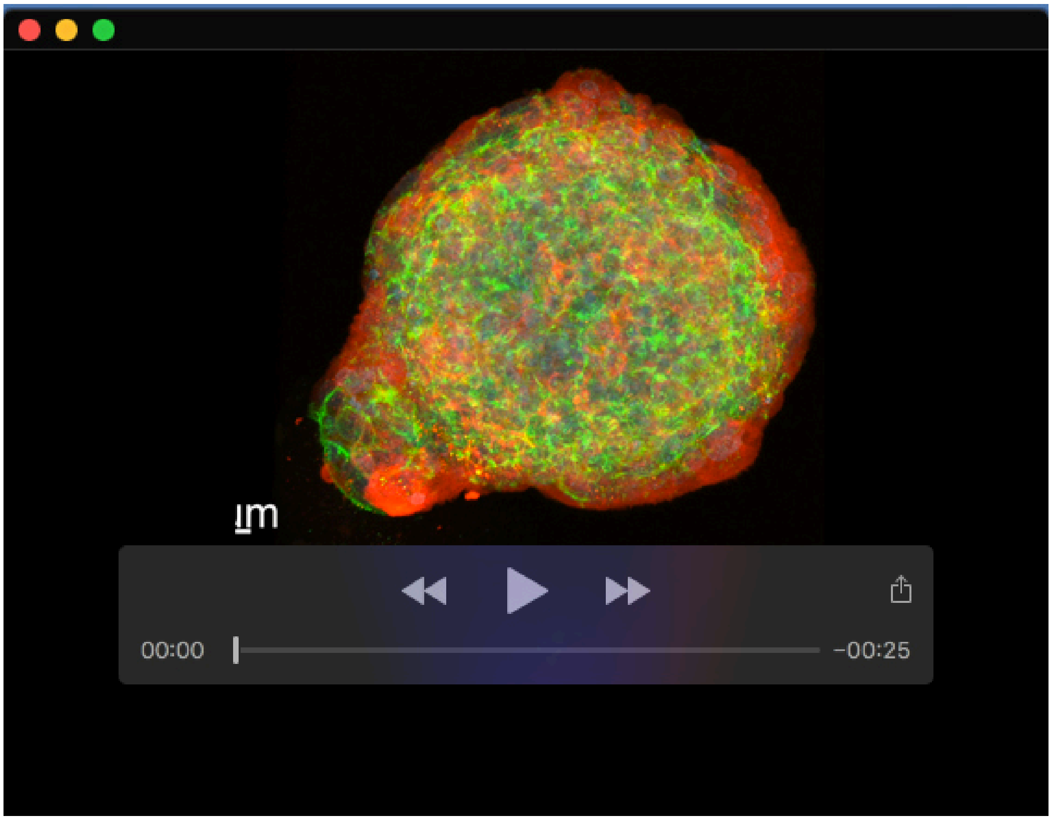

Movie 4. Three-dimensional image reconstruction of an STB^{out} trophoblast organoid (shown in Figure 2B, top) immunostained for ITGA6 (in green) and CGBs (in red). DAPI-stained nuclei are shown in grey.

Journal of Cell Science • Supplementary information
